# 1-De­oxy-d-arabinitol

**DOI:** 10.1107/S1600536808012555

**Published:** 2008-05-07

**Authors:** Sarah F. Jenkinson, Filipa P. Cruz, Kathrine V. Booth, George W. J. Fleet, Ken Izumori, Chu-Yi Yu, David J. Watkin

**Affiliations:** a Department of Organic Chemistry, Chemical Research Laboratory, University of Oxford, Mansfield Road, Oxford OX1 3TA, England; b Rare Sugar Research Centre, Kagawa University, 2393 Miki-cho, Kita-gun, Kagawa 761-0795, Japan; c Laboratory of Molecular Recognition and Selective Synthesis, Institute of Chemistry, Chinese Academy of Sciences, Beijing 10080, People’s Republic of China; d Department of Chemical Crystallography, Chemical Research Laboratory, University of Oxford, Mansfield Road, Oxford OX1 3TA, England

## Abstract

Addition of methyl lithium to d-erythrono-1,4-lactone followed by acid deprotection was shown, by X-ray crystallography, to give 1-de­oxy-d-arabinitol, C_5_H_12_O_4_, rather than 1-de­oxy-d-ribitol as the major product. The crystal structure exists as hydrogen-bonded chains of mol­ecules running parallel to the *c* axis which are further linked together by hydrogen bonds. Each mol­ecule is a donor and an acceptor for four hydrogen bonds.

## Related literature

For related literature see: Izumori (2002 ▶, 2006 ▶); Granstrom *et al.* (2004 ▶); Beadle *et al.* (1992 ▶); Skytte (2002 ▶); Levin (2002 ▶); Howling & Callagan (2000 ▶); Bertelsen *et al.* (1999 ▶); Takata *et al.* (2005 ▶); Menavuvu *et al.* (2006 ▶); Sui *et al.* (2005 ▶); Hossain *et al.* (2006 ▶); Zehner *et al.* (1994 ▶); Donner *et al.* (1999 ▶); Yoshihara *et al.* (2008 ▶); Takai & Heathcock (1985 ▶); Zissis & Richtmyer (1954 ▶).
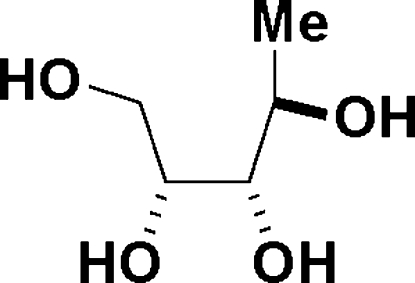


## Experimental

### 

#### Crystal data

C_5_H_12_O_4_
*M* *_r_* = 136.15
Tetragonal, 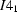

*a* = 12.9873 (5) Å
*c* = 8.3679 (3) Å
*V* = 1411.41 (9) Å^3^
*Z* = 8
Mo *K*α radiation
μ = 0.11 mm^−1^
*T* = 150 K
0.25 × 0.25 × 0.25 mm

#### Data collection

Nonius KappaCCD area-detector diffractometer
Absorption correction: multi-scan (*DENZO*/*SCALEPACK*; Otwinowski & Minor, 1997 ▶) *T*_min_ = 0.93, *T*_max_ = 0.97
3189 measured reflections
855 independent reflections
750 reflections with *I* > 2σ(*I*)
*R*_int_ = 0.020

#### Refinement

*R*[*F* ^2^ > 2σ(*F* ^2^)] = 0.043
*wR*(*F* ^2^) = 0.123
*S* = 1.00
855 reflections
82 parameters
1 restraint
H-atom parameters constrained
Δρ_max_ = 0.34 e Å^−3^
Δρ_min_ = −0.39 e Å^−3^

### 

Data collection: *COLLECT* (Nonius, 2001 ▶); cell refinement: *DENZO*/*SCALEPACK* (Otwinowski & Minor, 1997 ▶); data reduction: *DENZO*/*SCALEPACK*; program(s) used to solve structure: *SIR92* (Altomare *et al.*, 1994 ▶); program(s) used to refine structure: *CRYSTALS* (Betteridge *et al.*, 2003 ▶); molecular graphics: *CAMERON* (Watkin *et al.*, 1996 ▶); software used to prepare material for publication: *CRYSTALS*.

## Supplementary Material

Crystal structure: contains datablocks global, I. DOI: 10.1107/S1600536808012555/lh2622sup1.cif

Structure factors: contains datablocks I. DOI: 10.1107/S1600536808012555/lh2622Isup2.hkl

Additional supplementary materials:  crystallographic information; 3D view; checkCIF report

## Figures and Tables

**Table 1:** Hydrogen-bond geometry (Å, °)

*D*—H⋯*A*	*D*—H	H⋯*A*	*D*⋯*A*	*D*—H⋯*A*
O8—H8⋯O8^i^	0.96	1.76	2.698 (4)	164
O6—H6⋯O6^ii^	1.00	1.98	2.712 (4)	128
O4—H4⋯O1^iii^	0.98	1.77	2.718 (4)	162
O1—H1⋯O4^iv^	1.05	2.03	2.712 (3)	120

Symmetry codes: (i) 
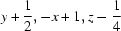
; (ii) 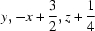; (iii) 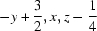; (iv) 
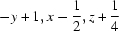
.

**Fig. 1.**
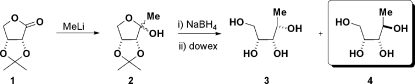
Synthetic scheme.

**Fig. 2.**
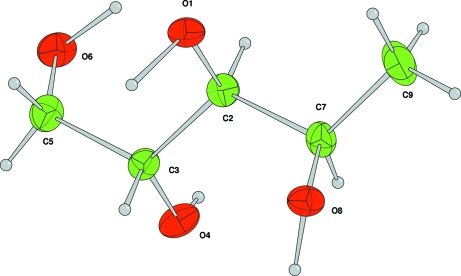
The title compound with displacement ellipsoids drawn at the 50% probability level. H atoms are shown as spheres of arbitary radius.

**Fig. 3.**
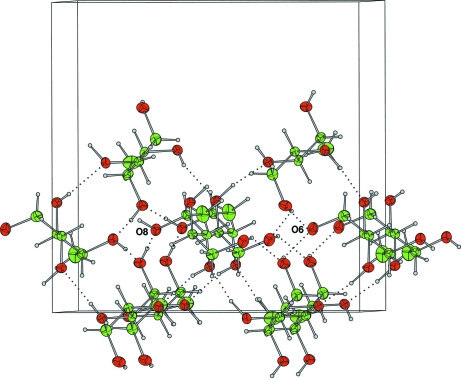
Packing diagram showing the O6—H6···O6 and O8—H8···O8 hydrogen bonds.

**Fig. 4.**
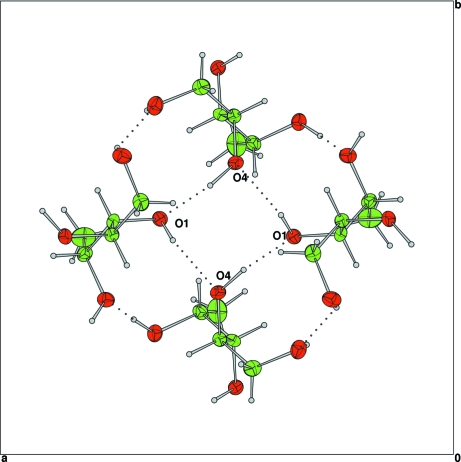
Packing diagram showing the O1—H1···O4—H4···O1 hydrogen bonds.

**Fig. 5.**
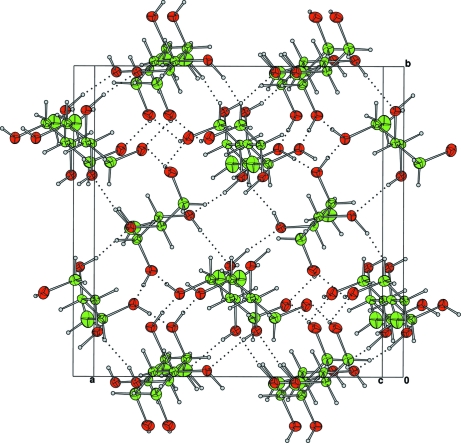
Packing diagram for the compound projected along the c-axis. Each molecule is a donor and an acceptor for 4 hydrogen-bonds.

## supplementary crystallographic information

### Comment

The demand for the large scale production of rare sugars by biotechnological (Izumori, 2006; Izumori, 2002; Granstrom *et al.*, 2004) and chemical (Beadle *et al.*, 1992) methods is driven by the demand for alternative foodstuffs (Skytte, 2002) and D-tagatose itself is used as a low calorie sweetener (Levin, 2002; Howling & Callagan, 2000; Bertelsen *et al.* 1999) Rare monosaccharides have been found to demonstrate interesting pharmaceutical properties, for example, D-psicose (Takata *et al.*, 2005; Menavuvu *et al.*, 2006) and D-allose (Sui *et al.*, 2005; Hossain *et al.*, 2006) have significant chemotherapeutic properties and D-tagatose has been found to be an anti-hyperglycemic agent (Zehner *et al.*, 1994; Donner *et al.*, 1999) and therefore potentially useful in the treatment of diabetes.

The methodology developed by Izumori *et al.* (2002, 2006) for the interconversion of tetroses, pentoses and hexoses by enzymatic oxidation, inversion at C3 with a single epimerase, and reduction to the aldose has been seen to be generally applicable for the 1-deoxy ketohexoses (Yoshihara *et al.*, 2008). In order to investigate the viability of this process to the corresponding pentoses and thus to evaluate their therapeutic potential 1-deoxy-D-arabinitol was synthesized, in 3 steps, from 2,3-*O*-isopropylidene-D-erythronolactone 1 (Fig.1). It has previously been seen that the four diastereomeric tetraols are very difficult to distinguish between by NMR spectroscopy (Takai & Heathcock, 1985). X-ray crystallography confirmed that the major product was the arabinitol 4 rather than the ribitol 3 which differs only in the stereochemistry at the C2 position (Fig. 2).

The molecules are linked by three hydrogen bonding systems and the structure consists of alternating spiral chains of O6—H6···O6 or O8—H8···O8 hydrogen-bonded molecules running parallel to the *c*-axis (Fig. 3) interconnected by O1—H1···O4—H4···O1 hydrogen bonds (Fig.4). Each molecule is a donor and acceptor for 4 hydrogen bonds (Fig. 5).

In summary, the stereochemistry at C2 of the title compound 1-deoxy-D-arabinitol 4 was firmly established by X-ray crystallography, the absolute configuration is determined by the use of D-erythronolactone as the starting material. As well as the potential biological properties of 1-deoxy ketoses, they are likely to provide a new set of building blocks for the synthesis of a wide variety of complex biomolecules.

### Experimental

The title compound was recrystallized from hot methanol: m.p. 398–400 K; [α]_D_^21^ +0.8 (*c*, 8 in H_2_O) {Lit. (Zissis & Richtmyer, 1954) m.p. 129–131°C; [α]_D_^20^ +0.7 (*c*, 10 in H_2_O; *l*, 4)}.

### Refinement

In the absence of significant anomalous scattering, Friedel pairs were merged and the absolute configuration assigned from the starting material.

The H atoms were all located in a difference map, but those attached to carbon atoms were repositioned geometrically. The H atoms were initially refined with soft restraints on the bond lengths and angles to regularize their geometry (C—H in the range 0.93–0.98, O—H = 0.82 Å) and *U*_iso_(H) (in the range 1.2–1.5 times *U*_eq_ of the parent atom), after which the positions were refined with riding constraints.

### Crystal data

**Table:**

C_5_H_12_O_4_	*Z* = 8
*M**_r_* = 136.15	*F*_000_ = 592
Tetragonal, *I*4_1_	*D*_x_ = 1.281 Mg m^−^^3^
Hall symbol: I 4bw	Mo *K*α radiation λ = 0.71073 Å
*a* = 12.9873 (5) Å	Cell parameters from 815 reflections
*b* = 12.9873 (5) Å	θ = 5–27º
*c* = 8.3679 (3) Å	µ = 0.11 mm^−^^1^
α = 90º	*T* = 150 K
β = 90º	Block, colourless
γ = 90º	0.25 × 0.25 × 0.25 mm
*V* = 1411.41 (9) Å^3^	

### Data collection

**Table:**

Nonius KappaCCD area-detector diffractometer	750 reflections with *I* > 2σ(*I*)
Monochromator: graphite	*R*_int_ = 0.020
*T* = 150 K	θ_max_ = 27.5º
ω scans	θ_min_ = 5.3º
Absorption correction: multi-scan(DENZO/SCALEPACK; Otwinowski & Minor, 1997)	*h* = −16→16
*T*_min_ = 0.93, *T*_max_ = 0.97	*k* = −11→11
3189 measured reflections	*l* = −10→10
855 independent reflections	

### Refinement

**Table:**

Refinement on *F*^2^	Primary atom site location: structure-invariant direct methods
Least-squares matrix: full	Hydrogen site location: inferred from neighbouring sites
*R*[*F*^2^ > 2σ(*F*^2^)] = 0.043	H-atom parameters constrained
*wR*(*F*^2^) = 0.123	*w* = 1/[σ^2^(*F*^2^) + ( 0.07*P*)^2^ + 1.26*P*], where *P* = (max(*F*_o_^2^,0) + 2*F*_c_^2^)/3
*S* = 1.00	(Δ/σ)_max_ = 0.002
855 reflections	Δρ_max_ = 0.34 e Å^−^^3^
82 parameters	Δρ_min_ = −0.39 e Å^−^^3^
1 restraint	Extinction correction: None

### Fractional atomic coordinates and isotropic or equivalent isotropic displacement parameters (Å^2^)

**Table:**

	*x*	*y*	*z*	*U*_iso_*/*U*_eq_	
O1	0.64776 (13)	0.51955 (15)	0.6622 (3)	0.0211	
C2	0.75127 (18)	0.5139 (2)	0.6068 (4)	0.0186	
C3	0.7537 (2)	0.4842 (2)	0.4296 (4)	0.0187	
O4	0.85700 (13)	0.48073 (16)	0.3723 (3)	0.0237	
C5	0.6897 (2)	0.5564 (2)	0.3268 (4)	0.0235	
O6	0.73116 (15)	0.65798 (14)	0.3242 (3)	0.0250	
C7	0.8135 (2)	0.4417 (2)	0.7135 (4)	0.0208	
O8	0.76689 (14)	0.34124 (13)	0.7126 (3)	0.0216	
C9	0.8162 (3)	0.4788 (2)	0.8844 (4)	0.0371	
H21	0.7853	0.5822	0.6286	0.0184*	
H31	0.7208	0.4168	0.4126	0.0196*	
H51	0.6985	0.5315	0.2238	0.0277*	
H52	0.6191	0.5542	0.3475	0.0271*	
H71	0.8827	0.4379	0.6604	0.0259*	
H91	0.8413	0.4265	0.9544	0.0541*	
H92	0.8595	0.5396	0.8958	0.0548*	
H93	0.7474	0.4971	0.9202	0.0552*	
H1	0.6194	0.4722	0.5703	0.0308*	
H8	0.7975	0.2944	0.6379	0.0334*	
H6	0.7418	0.6761	0.4388	0.0359*	
H4	0.9070	0.5369	0.3651	0.0365*	

### Atomic displacement parameters (Å^2^)

**Table:**

	*U*^11^	*U*^22^	*U*^33^	*U*^12^	*U*^13^	*U*^23^
O1	0.0197 (10)	0.0197 (9)	0.0240 (12)	0.0010 (7)	0.0042 (9)	−0.0023 (9)
C2	0.0139 (13)	0.0193 (12)	0.0225 (16)	−0.0037 (9)	0.0006 (12)	−0.0001 (13)
C3	0.0176 (14)	0.0167 (12)	0.0218 (17)	−0.0006 (9)	−0.0011 (12)	0.0010 (13)
O4	0.0169 (9)	0.0213 (9)	0.0329 (14)	0.0015 (7)	0.0061 (10)	0.0035 (10)
C5	0.0223 (14)	0.0269 (15)	0.0214 (16)	0.0014 (11)	0.0013 (13)	0.0032 (15)
O6	0.0308 (11)	0.0215 (10)	0.0227 (12)	0.0025 (8)	0.0056 (11)	0.0046 (10)
C7	0.0201 (13)	0.0204 (13)	0.0218 (16)	−0.0035 (10)	−0.0045 (13)	−0.0004 (13)
O8	0.0254 (10)	0.0189 (10)	0.0204 (12)	0.0010 (7)	0.0049 (10)	0.0000 (9)
C9	0.053 (2)	0.0332 (16)	0.0253 (15)	−0.0023 (15)	−0.0149 (15)	−0.0036 (13)

### Geometric parameters (Å, °)

**Table:**

O1—C2	1.424 (3)	C5—H51	0.927
O1—H1	1.051	C5—H52	0.934
C2—C3	1.532 (3)	O6—H6	0.997
C2—C7	1.527 (4)	C7—O8	1.438 (3)
C2—H21	1.008	C7—C9	1.510 (5)
C3—O4	1.425 (3)	C7—H71	1.004
C3—C5	1.520 (4)	O8—H8	0.959
C3—H31	0.985	C9—H91	0.954
O4—H4	0.978	C9—H92	0.974
C5—O6	1.425 (3)	C9—H93	0.972
			
C2—O1—H1	93.6	C3—C5—H52	114.4
O1—C2—C3	110.4 (2)	O6—C5—H52	113.8
O1—C2—C7	109.9 (3)	H51—C5—H52	106.4
C3—C2—C7	113.6 (2)	C5—O6—H6	104.9
O1—C2—H21	108.0	C2—C7—O8	109.4 (2)
C3—C2—H21	112.9	C2—C7—C9	111.7 (2)
C7—C2—H21	101.7	O8—C7—C9	107.7 (3)
C2—C3—O4	110.7 (2)	C2—C7—H71	104.2
C2—C3—C5	112.4 (2)	O8—C7—H71	109.3
O4—C3—C5	110.1 (2)	C9—C7—H71	114.4
C2—C3—H31	110.8	C7—O8—H8	113.9
O4—C3—H31	109.4	C7—C9—H91	111.2
C5—C3—H31	103.2	C7—C9—H92	111.4
C3—O4—H4	128.4	H91—C9—H92	108.7
C3—C5—O6	111.9 (2)	C7—C9—H93	110.4
C3—C5—H51	104.0	H91—C9—H93	107.4
O6—C5—H51	105.2	H92—C9—H93	107.6

### Hydrogen-bond geometry (Å, °)

**Table:**

*D*—H···*A*	*D*—H	H···*A*	*D*···*A*	*D*—H···*A*
O8—H8···O8^i^	0.96	1.76	2.698 (4)	164
O6—H6···O6^ii^	1.00	1.98	2.712 (4)	128
O4—H4···O1^iii^	0.98	1.77	2.718 (4)	162
O1—H1···O4^iv^	1.05	2.03	2.712 (3)	120

Symmetry codes: (i) *y*+1/2, −*x*+1, *z*−1/4; (ii) *y*, −*x*+3/2, *z*+1/4; (iii) −*y*+3/2, *x*, *z*−1/4; (iv) −*y*+1, *x*−1/2, *z*+1/4.
